# Impact of race and ethnicity on clinical outcomes and recurrence post‐ureteral reconstruction

**DOI:** 10.1002/bco2.450

**Published:** 2024-11-07

**Authors:** Dhruv Puri, Eric Cho, Kian Ahmadieh, Nishant Garg, Cesar Delgado, Benjamin Cedars, Michael Witthaus, Michael Pan, Jill C. Buckley

**Affiliations:** ^1^ Department of Urology UC San Diego School of Medicine La Jolla California USA; ^2^ Kaiser Permanente, Redwood City Medical Center San Francisco California USA; ^3^ University of Maryland Capital Region Medical Center Baltimore Maryland USA; ^4^ Kelsey‐Seybold Clinic Houston Texas USA

**Keywords:** health disparities, race and ethnicity, robot‐assisted ureteroplasty, ureteral stricture disease

## Abstract

**Introduction:**

Ureteral stricture disease (UTSD) poses significant challenges in reconstructive urology, with recent advances highlighting disparities in healthcare outcomes based on race and ethnicity. This study investigates the impact of race and ethnicity on clinical outcomes following ureteral reconstruction.

**Methods:**

We conducted a single‐centre prospective analysis of 233 patients who underwent ureteral reconstruction for UTSD from 2014 to 2023. Patient demographics, clinical characteristics, surgical details and outcomes were collected. Patients were stratified by race (White vs. non‐White) and ethnicity (Hispanic vs. non‐Hispanic). Statistical analyses included Kruskal–Wallis, Mann–Whitney *U* tests, ANOVA, Kaplan–Meier analysis and multivariate logistic regression.

**Results:**

Our cohort included 233 patients who underwent ureteroplasty with 108 (46.4%) non‐White patients, and 71 (30.5%) were Hispanic. No significant differences were found in recurrence rates, complications, or stricture‐free survival between racial and ethnic groups. Prior reconstructions were more prevalent among non‐White patients (26.9% vs. 16.0%; *p* = 0.043). Unadjusted and adjusted regressions showed significant associations between non‐White race (unadjusted *β* = 0.76, *p* = 0.008; adjusted *β* = 0.82, *p* = 0.008) and Hispanic ethnicity (unadjusted *β* = 0.70, *p* = 0.025; adjusted *β* = 0.79, *p* = 0.020) with increased stricture lengths.

**Conclusion:**

This study highlights that although recurrence and complication rates do not significantly differ by race or ethnicity, disparities exist in clinical presentations, with non‐White and Hispanic patients presenting with longer stricture lengths and higher body mass index. These findings underscore the need for targeted interventions to address underlying disparities in healthcare delivery and access.

## 1 INTRODUCTION

Ureteral stricture disease (UTSD) represents one of the most evolving challenges for the field of reconstructive urology.^1^ Lack of treatment can result in serious repercussion including chronic pain, voiding dysfunction, infection, fistulae and renal failure.^2^ Recent advances in health equity have identified significant disparities in urologic care with regards to race and ethnicity.^3^, ^4^, ^5^, ^6^ A recent single‐institution study found no significant difference in the rate of recurrence after surgical repair for urethral stricture based on race.^7^ However, with respect to upper tract disease, such disparities have yet to be explored.

Race, ethnicity and cultural background can significantly influence healthcare outcomes, particularly in urology.^3^, ^5^, ^8^, ^9^ For example, urolithiasis and urinary tract infections (UTIs) are conditions where these factors may play a critical role in the development and worsening of UTSD.^10^, ^11^ Disparities in access to care can lead to higher stone burdens, resulting in more invasive and more staged procedures for treating urolithiasis.^6^, ^12^ Additionally, studies have shown that lower socioeconomic status and the use of an interpreter are associated with a significantly increased risk of multidrug‐resistant UTIs, complicating treatment outcomes.^13^ Minority groups, particularly Black and Hispanic populations, are at higher risk for infections with pathogens like *Escherichia coli*, further exacerbating disparities in urologic care.^14^ Understanding these nuances is crucial for tailoring interventions and improving outcomes in diverse patient populations. As such, we sought to investigate the prevalence and impact of race and ethnicity on outcomes in patients with UTSD following ureteral reconstructive surgery, specifically aiming to assess whether these risk factors remained consistent in upper tract ureteral reconstruction within our diverse social ecosystem.

## 2 METHODS

### 2.1 Study population

We conducted a single‐centre, prospective data collection with retrospective review of all patients undergoing ureteral reconstruction from 2016 to 2023. Inclusion criteria for the study required patients to have undergone ureteral reconstruction with a minimum follow‐up interval of 3 months from the time of surgery. Institutional Review Board approval was obtained (IRB 460337). Patients who exhibited clinical manifestations of UTSD were managed and monitored by a reconstructive urology team, adhering strictly to the most current clinical management guidelines. It is noteworthy that all surgical procedures were meticulously carried out by a seasoned surgeon with proficiency in both open and robot‐assisted surgical techniques, contributing to the consistency and reliability of the surgical outcomes documented in this analysis.

### 2.2 Data collection

Patient demographic, clinical and disease features were recorded. Demographics included patient sex, race, ethnicity, age at diagnosis, body mass index (BMI, kg/m^2^) at diagnosis, history of smoking and history of diabetes mellitus or chronic kidney disease. Clinical disease includes prior endoscopic management (endopyelotomy), history of pelvic radiation, history of prior reconstructive urologic surgery, type of ureteral reconstruction (pyeloplasty, reimplant, ileal ureter or ureteroureterostomy) and operating room time (minutes). Stricture characteristics included stricture laterality (left, right, bilateral and transplant), stricture length (<2 cm, >2 cm and continuous) and stricture location (proximal: renal pelvis to the start of the sacroiliac joint [L2–L3/L4]; middle: across the SI joint [L4–S1]; distal: below the SI joint within the pelvis [below S1]; proximal + mid; mid + distal; pan‐ureteral). The primary outcome of interest was recurrence and secondary outcomes were 30‐day UTI rate, 30‐day complication (Clavien > 2) rate, 30‐day readmission rate and if the patient was symptomatic at the last follow up. Recurrence was defined as follows: prevalence upon imaging or requiring subsequent intervention (stent, percutaneous nephrostomy or further reconstructive procedure).

### 2.3 Statistical analysis

We stratified patients by race and ethnicity into two comparison cohorts: White compared with all other races and Hispanic compared with Non‐Hispanic. Descriptive analyses were conducted utilizing Kruskall–Wallis test (nonparametric one‐way ANOVA) and Mann–Whitney *U* tests for categorical variables and ANOVA for continuous variables. Kaplan–Meier analysis evaluated the differences in stricture‐free rate for different race and ethnicity groupings. Logistic regression multivariable analyses elucidated predictors for stricture length. SPSS® v.27 (IBM®, Armonk, New York, USA) were utilized for statistical analyses, with a predetermined *p* < 0.05 considered the threshold for significance.

## 3 RESULTS

Our cohort analysis encompassed 233 patients subjected to ureteral reconstruction for UTSD over 2015–2023. The racial breakdown included 125 White patients (53.6%), 22 Asian (9.4%), 5 Black (2.1%), 2 American Indian or Alaska Native (0.9%), and 79 (33.8%) classified as ‘other’. Ethnicity‐wise, the cohort comprised 71 Hispanic (30.5%) and 162 non‐Hispanic (69.5%) patients. The mean age was 53.7 years with a median of 56 years. There were 54 obese patients (23.2%), 87 with a history of smoking (37.3%) and a median follow‐up duration of 12 months (interquartile range 5–24).

Racial demographics and disease features are condensed in Table 1. White patients had a longer median follow‐up time of 13 months versus 12 months in non‐White patients. Non‐White patients had a lower mean age at diagnosis (49.3 years vs. 57.3 years; *p* < 0.001) and a higher mean BMI (28.0 vs. 25.8; *p* = 0.002). Prior reconstructions were more prevalent among non‐White patients (26.9% vs. 16.0%; *p* = 0.043) while a history of pelvic radiation was less common (2.8% vs. 12.8%; *p* = 0.005). Rates of prior endoscopic management were not significantly different (*p* = 0.061), and no disparities were detected in ureteral reconstruction type (*p* = 0.121). Stricture length was notably longer in non‐White patients (2.93 cm vs. 2.17 cm; *p* = 0.019). No significant differences were observed in stricture laterality (*p* = 0.971), location (*p* = 0.484), recurrence rates (*p* = 0.575), follow‐up duration (*p* = 0.351), 30‐day UTI rates (*p* = 0.633), complications (*p* = 0.341) or readmissions (*p* = 0.807).

**TABLE 1:** Demographics, clinical disease characteristics and outcomes stratified by race.

	White (*n* = 125)	Non‐White (*n* = 108)	*p* value
Sex			0.491
Male	67 (53.6)	53 (49.1)	
Female	58 (46.4)	55 (50.9)	
Age at diagnosis	57.31 (59)	49.31 (55)	< 0.001
BMI at diagnosis	25.8 (25.2)	28.0 (27.0)	0.002
Smoking			0.503
Active smoker	12 (9.6)	13 (12.0)	
Former smoker	37 (29.6)	25 (23.1)	
Never smoked	76 (60.8)	70 (64.8)	
History of diabetes	11 (8.8)	15 (13.9)	0.219
History of CKD	23 (18.4)	14 (13.0)	0.257
Prior endoscopic management	39 (31.2)	22 (20.4)	0.061
Prior ureteral reconstruction	20 (16.0)	29 (26.9)	0.043
History of pelvic radiation	16 (12.8)	3 (2.8)	0.005
Type of surgery			
Pyeloplasty	49 (39.2)	46 (42.6)	0.121
Reimplant	67 (53.6)	52 (48.1)	
Ileal ureter	9 (7.2)	6 (5.6)	
Ureteroureterostomy	0 (0.0)	4 (3.7)	
OR time (minutes)	227.0 (201)	228.9 (217)	0.451
Stricture laterality			
Left	60 (48.0)	54 (50.0)	0.971
Right	54 (43.2)	44 (40.7)	
Bilateral	4 (3.2)	3 (2.8)	
Transplant	7 (5.6)	7 (6.5)	
Stricture length (cm)	2.17 (2)	2.93 (2.3)	0.004
Stricture location			
Proximal	59 (47.2)	55 (50.9)	0.089
Middle	7 (5.6)	9 (8.3)	
Distal	48 (38.4)	32 (29.6)	
Proximal + mid	1 (0.8)	2 (1.9)	
Mid + distal	3 (2.4)	1 (0.9)	
Pan‐ureteral	0 (0.0)	1 (0.9)	
Follow‐up time in months	18.15 (13)	17.2 (12)	0.351
30‐day UTI	18 (14.4)	18 (16.7)	0.633
30‐day complication (Clavien > 2)	27 (21.6)	18 (16.7)	0.341
30‐day readmission	16 (12.8)	15 (13.9)	0.807
Symptom severity at last follow up			
Symptomatic (better than preop)	7 (5.6)	13 (12.0)	0.164
Symptomatic (similar to preop)	2 (1.6)	0 (0.0)	
Symptomatic (worse than preop)	3 (2.4)	4 (3.7)	
Asymptomatic	113 (90.4)	91 (84.3)	
Stricture recurrence	7 (5.6)	8 (7.4)	0.575

Ethnicity‐related demographics and disease features are collated in Table 2. The median follow‐up for Hispanic patients was 13 months, with a range up to 74 months, while non‐Hispanic patients had a median of 12 months, with a range up to 115 months. Higher BMI at diagnosis was observed in Hispanic patients (29.8 vs. 25.5; *p* < 0.001), along with a greater history of diabetes (19.7% vs. 7.4%; *p* = 0.006). No significant differences were reported in prior endoscopic management (*p* = 0.849), reconstruction types (*p* = 0.275), stricture laterality (*p* = 0.431) or location (*p* = 0.050). Similar to the racial data, recurrence rates (*p* = 0.804), follow‐up time (*p* = 0.368), 30‐day UTI rates (*p* = 0.438), complications (*p* = 0.537) and readmissions (*p* = 0.852) did not differ significantly.

**TABLE 2:** Demographics, clinical disease characteristics and outcomes stratified by ethnicity.

	Hispanic (*n* = 71)	Non‐Hispanic (*n* = 162)	*p* value
Sex			0.31
Male	33 (46.5)	87 (53.7)	
Female	38 (53.5)	75 (46.3)	
Age at diagnosis	50.9 (56)	54.8 (56)	0.051
BMI at diagnosis	29.8 (29)	25.5 (25)	< 0.001
Smoking			0.275
Active smoker	9 (12.7)	16 (9.9)	
Former smoker	14 (19.7)	48 (29.6)	
Never smoked	48 (67.6)	98 (60.5)	
History of diabetes	14 (19.7)	12 (7.4)	0.006
History of CKD	10 (14.1)	27 (16.7)	0.62
Prior endoscopic management	18 (25.4)	43 (26.5)	0.849
Prior ureteral reconstruction	20 (28.2)	29 (17.9)	0.077
History of pelvic radiation	2 (2.8)	17 (10.5)	0.049
Type of surgery			0.275
Pyeloplasty	26 (36.6)	69 (42.6)	
Reimplant	38 (53.5)	81 (50.0)	
Ureteroureterostomy	4 (5.6)	11 (6.8)	
Ileal ureter	3 (4.2)	1 (0.6)	
OR time in minutes	227.3 (217)	228.1 (205.5)	0.481
Stricture laterality			
Left	40 (56.3)	74 (45.7)	0.431
Right	26 (36.6)	72 (44.4)	
Bilateral	1 (1.4)	6 (3.7)	
Transplant	4 (5.6)	10 (6.2)	
Stricture length	2.97 (2)	2.27 (2)	0.012
Stricture location			
Proximal	34 (47.9)	80 (49.4)	0.050
Middle	4 (5.6)	12 (7.4)	
Distal	26 (36.6)	54 (33.3)	
Proximal + mid	3 (4.2)	0 (0.0)	
Mid + distal	0 (0.0)	4 (2.4)	
Pan‐ureteral	1 (1.4)	0 (0.0)	
Follow‐up time in months	17.1 (13)	18.0 (12)	0.368
30‐day UTI	9 (12.7)	27 (16.7)	0.438
30 day complication (Clavien > 2)	12 (16.9)	33 (20.4)	0.537
30‐day readmission	9 (12.7)	22 (13.6)	0.852
Symptom severity at last follow up			
Symptomatic (better than preop)	5 (7.0)	15 (9.3)	0.388
Symptomatic (similar to preop)	1 (1.4)	1 (0.6)	
Symptomatic (worse than preop)	4 (5.6)	3 (1.9)	
Asymptomatic	61 (85.9)	143 (88.3)	
Stricture recurrence	5 (7.0)	10 (6.2)	0.803

**FIGURE 1:**
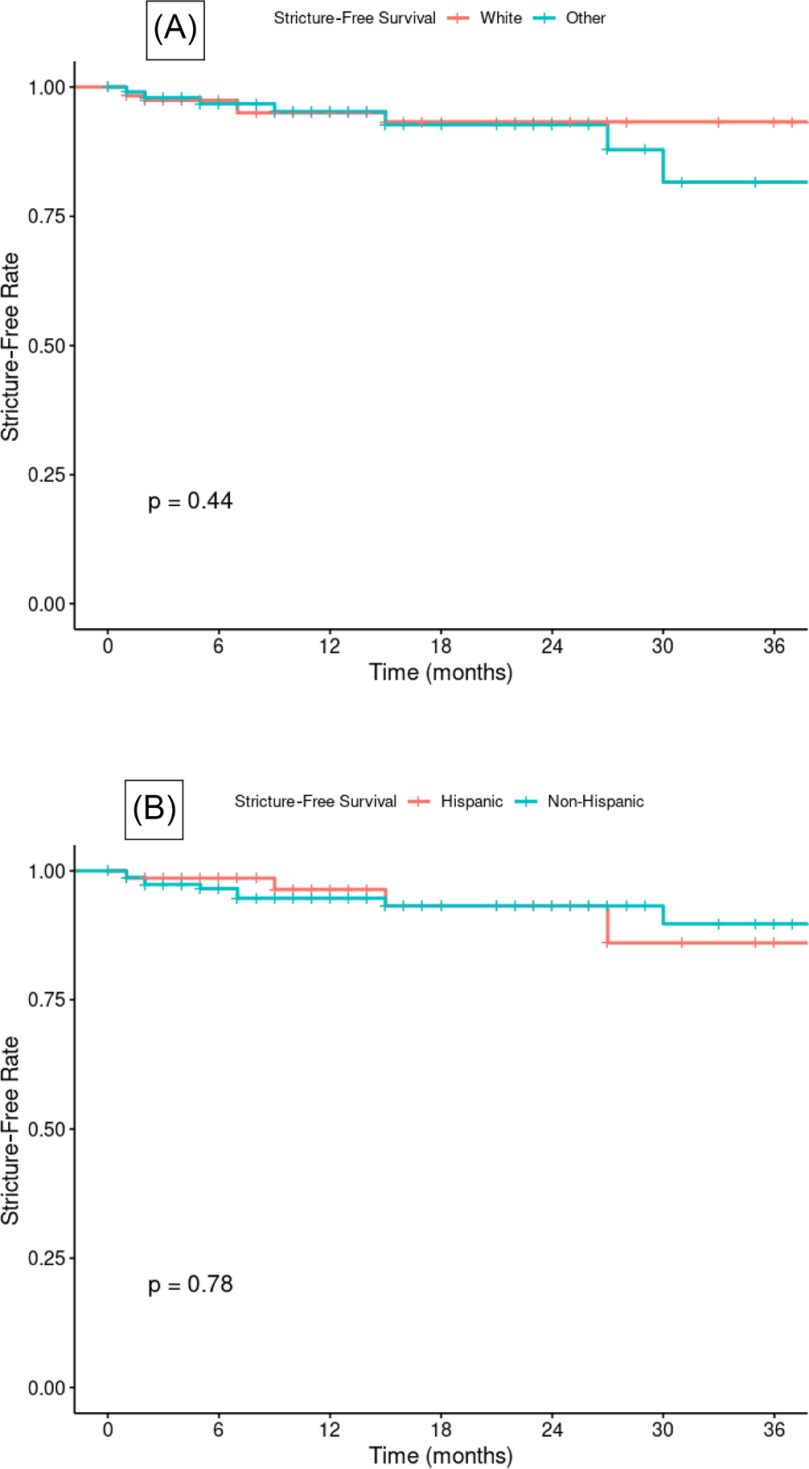
The Kaplan–Meier analyses comparing stricture‐free survival by (A) race (B) ethnicity.

Recurrent ureteral strictures were documented in 15 (6.4%) patients. Management varied: additional reconstructive surgery in 7 cases, chronic percutaneous nephrostomy (PCN) or stent maintenance in 7, and 1 was lost to follow‐up. Kaplan–Meier curves (Figure 1A,B) demonstrated no significant differences in stricture‐free survival between racial (*p* = 0.44) or ethnic groups (*p* = 0.78), with median months to recurrence being 7 for White versus 12 for non‐White (*p* = 0.251) and 15 for Hispanic versus 6 for non‐Hispanic (*p* = 0.193).

Table 3 provides a covariate‐adjusted linear regression analysis assessing race and ethnicity's impact on stricture length, controlling for age at diagnosis, BMI and prior endoscopic management. Both unadjusted and adjusted regressions showed significant associations between non‐White race (unadjusted *β* = 0.76, *p* = 0.008; adjusted *β* = 0.82, *p* = 0.008) and Hispanic ethnicity (unadjusted *β* = 0.70, *p* = 0.025; adjusted *β* = 0.79, *p* = 0.020) with increased stricture lengths.

**TABLE 3:** Univariate and multivariate linear regression for stricture length.

Variable	Beta (*β*)	*p* value
Univariate analysis		
Non‐White vs. White	0.76 (0.20–1.32)	0.008
Hispanic vs. non‐Hispanic	0.697 (0.09–1.30)	0.025
Multivariate analysis		
Non‐White vs white	0.816 (0.22–1.42)	0.008
Increasing BMI at diagnosis	−0.01 (−0.06 to 0.04)	0.805
Increasing age at diagnosis	0.01 (−0.01 to 0.02)	0.591
Prior endoscopy management	0.142 (−0.46 to 0.74)	0.64
Hispanic vs non‐Hispanic	0.793 (0.12–1.46)	0.02
Increasing BMI at diagnosis	−0.011 (−0.06 to 0.04)	0.678
Increasing age at diagnosis	0.004 (−0.01 to 0.02)	0.66
Prior endoscopy management	0.202 (−0.04 to 0.81)	0.511

## 4 DISCUSSION

Our study provides the first ever exploration of race and ethnicity in the context of UTSD. Overall, within this cohort of 233 patients, 33.8% were non‐White and 30.5% Hispanic. No significant differences emerged across racial or ethnic lines in terms of stricture characteristics, recurrence rates or postoperative complications. Non‐White patients presented with a lower mean age and higher BMI compared with White patients and were more likely to have undergone prior reconstructions but less likely to have had pelvic radiation. Regarding ethnicity, Hispanic patients had a higher BMI, and a greater number had a history of diabetes. Recurrence of ureteral strictures occurred in 6.4% of patients, and Kaplan–Meier analysis showed no substantial difference in stricture‐free survival between races or ethnicities. Adjusted linear regression analysis indicated that both non‐White race and Hispanic ethnicity were significantly associated with longer stricture lengths after accounting for age, BMI and history of dilation/DVIU.

The central aim of our investigation was to explore outcome disparities among patients undergoing upper tract reconstructive surgeries. Racial and ethnic differences have been implicated in various healthcare dimensions, including access to services, disease burden and surgical results.^3^, ^5^, ^6^ In the context of reconstructive surgery, Sawyer et al. analysed 307 undergoing urethroplasty and found no significant difference in the rate of surgical failure repair based on race. Anger et al also found that upon review of Medicare claims data, stricture prevalence was greatest among Black and Hispanic men.^15^

Our findings build on this conclusion: that postoperatively, non‐White and Hispanic patients exhibit stricture‐free survival and complication rates comparable with their White and non‐Hispanic counterparts. Although previous studies have pinpointed non‐diabetic status and robot‐assisted procedures as predictors of successful ureteroplasty outcomes, the potential impact of demographic variables has not been explored.^16^, ^17^ Despite no differences in stricture recurrence, a disproportionately higher number of non‐White patients presented to our hospital with a failed prior reconstruction. We believe that this may signal the presence of disparities in the delivery of care for non‐White patients in their stricture work up outside of a large academic centre with a reconstructive urology department. However, other aspects that contribute existing disparities may not have been captured in this analysis.

The geographical location of our hospital, serving a population at the US–Mexico border, adds a distinctive context to our findings of longer ureteral strictures in Hispanic and non‐White patients prior to surgery. This region has been consistently linked with healthcare disparities, including in urologic outcomes, where access to care and quality of treatment are often compromised.^18^, ^19^ These disparities are rooted in a complex interplay of factors such as socioeconomic status, healthcare infrastructure, cultural beliefs and possibly immigration‐related issues, which all can hinder timely and effective urologic care. For example, economic barriers and cross‐border healthcare navigation complexities can lead to delayed presentations and advanced disease states.^18^, ^20^, ^21^ Additionally, cultural factors, including varying health‐seeking behaviours and attitudes toward medical intervention, may further contribute to the delay in seeking care, thus exacerbating the severity of conditions by the time of clinical intervention.^22^, ^23^ It is essential to consider such regional and demographic nuances, as they offer valuable insights into the barriers faced by these communities. Understanding and addressing these localized healthcare delivery challenges is critical not only for enhancing surgical outcomes but also for ensuring that the success of surgical interventions can be shared equitably across different population groups.

One of our study's findings, the observed pattern of Hispanic and non‐White patients having longer ureteral strictures at the time of presentation for reconstructive surgery suggests several underlying dynamics that warrant cautious interpretation. Socioeconomic obstacles, cultural influences and healthcare system nuances might contribute to a delayed diagnosis and treatment, thus possibly allowing for disease progression. Of note is that the current literature is mixed on the predictive capacity pre‐intervention stricture length on outcomes, adding another layer of complexity to our understanding of these disparities.^24^, ^25^ Additionally, implicit biases and disparities in the distribution of healthcare services may also influence the stage at which these populations receive surgical care. These factors collectively highlight the complexity of healthcare delivery and the necessity of adopting a multi‐faceted approach to understanding and ultimately mitigating these disparities. Thus, our conclusions here are preliminary, and we acknowledge the need for further research to clarify these associations and inform targeted interventions.

This study is not without limitations. First, our analysis is constrained by its retrospective nature and the single‐centre design, which may introduce selection bias and limit the generalizability of our findings to other populations or healthcare settings. The cohort's racial and ethnic diversity, although comprehensive for our institution, may not reflect the broader demographic spectrum, particularly with the underrepresentation of certain racial groups. Furthermore, the reliance on medical records for data extraction can lead to information bias, given the potential variability in documentation practices and clinical evaluations. Additionally, the observational design precludes the establishment of causal relationships between race, ethnicity, and UTSD outcomes. There is also the possibility of residual confounding, despite attempts to adjust for known confounders in our regression analyses. Moreover, the absence of significant differences in several outcomes, such as recurrence rates and complications, might be attributable to a type II error, considering the relatively small sample size for certain subgroups. The follow‐up period, although sufficient to detect immediate postoperative outcomes, may not be long enough to capture long‐term complications or late recurrences, which are particularly relevant in the context of ureteral reconstructive surgery. Lastly, our study did not consider other socioeconomic factors like insurance coverage, years living in the united states, specific location of diagnosis or access to care, which are critical factors that can influence healthcare outcomes and could contribute to observed disparities. Future multicentre, prospective studies are needed to overcome these limitations, enhance robustness and provide a more comprehensive understanding of the impact of race and ethnicity on UTSD outcomes.

## 5 CONCLUSION

Our comprehensive study serves as the first investigation into how racial and ethnic backgrounds interplay with the presentation and management of UTSD. Recurrence of ureteral strictures was documented in a minor fraction of 6.4% of the cohort, with stricture‐free survival rates and postop complications showing no substantial variation between the different racial and ethnic groups, as indicated by Kaplan–Meier analysis. An adjusted linear regression suggested a significant association of non‐White race and Hispanic ethnicity with extended lengths of stricture. We observed different metrics that may indicate existing disparities including stricture length, length of follow up and failed prior reconstruction between racial and ethnic groups that warrant future studies.

## AUTHOR CONTRIBUTIONS

*Conceptualization*: Dhruv Puri, Kian Ahmadieh, and Jill C. Buckley. *Data curation*: Dhruv Puri, Eric Cho, Kian Ahmadieh, Ben Cedars, Nishant Garg, Cesar Delgado, Michael Witthaus and Michael Pan. *Formal analysis*: Dhruv Puri. *Funding acquisition*: Jill C. Buckley. *Investigation*: Jill C. Buckley. *Methodology*: Dhruv Puri, Eric Cho, Ben Cedars, Michael Witthaus and Michael Pan. *Project administration*: Jill C. Buckley, Michael Pan and Michael Witthaus. *Resources*: Michael Pan and Cesar Delgado. *Software*: Dhruv Puri and Eric Cho. *Supervision*: Jill C. Buckley. *Validation*: Jill C. Buckley. *Writing the original draft*: Dhruv Puri, Erich Cho, Kian Ahmadieh and Eric Cho. *Writing review and editing*: all authors. All authors provided critical feedback and helped shape the research, analysis and manuscript. All authors read and approved the final manuscript.

## CONFLICT OF INTEREST STATEMENT

All authors declare no conflicts exist.
